# Supplementary material for: Molecular Pathways Associated with Cold Tolerance in Grafted Cucumber (Cucumis sativus L.)
Source: Plants (Basel). 2025 Dec 18;14(24):3860. doi: 10.3390/plants14243860 (PMC12736856; doi:10.3390/plants14243860)
Supplement: Supplementary file 1 [file plants-14-03860-s001.zip › Additional file S1_plants_revised.pdf]

plants

Supplementary file

Molecular pathways associated with cold tolerance in grafted cucumber (*Cucumis sativus* L.)

Sudeep Pandey[1,2], Bijaya Sharma Subedi[1], and Andrew B. Ogden[1,2]*

[1] Department of Horticulture, University of Georgia, 1109 Experiment Street, Griffin, GA, 30223, USA.
[2] Institute of Plant Breeding, Genetics, & Genomics, 1109 Experiment Street, Griffin, GA, 30223, USA.
* Correspondence: aogden@uga.edu

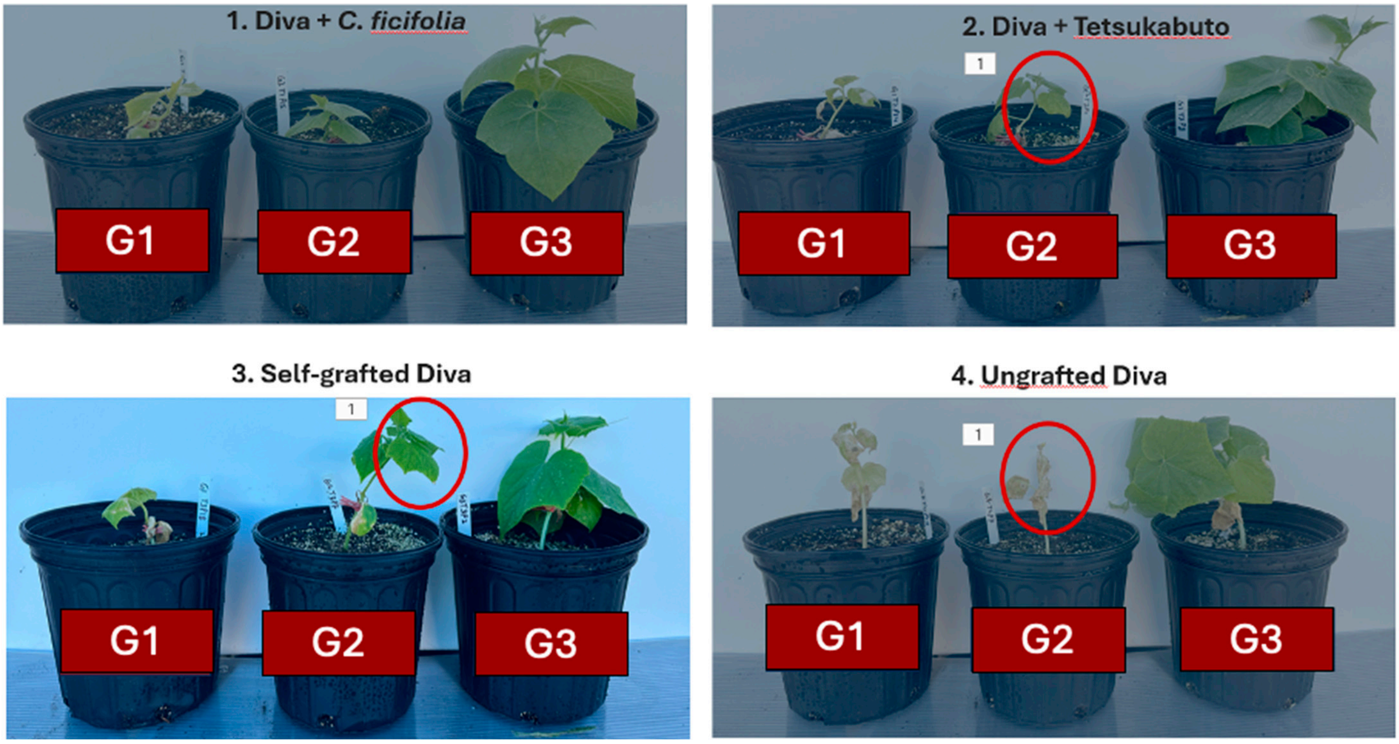

Figure S1: Severity of grafted and ungrafted plants at three different growth chambers G1 (12/7°C), G2 (18/12°C), and G3 (24/18°C) after 21 days.

 https://doi.org/10.3390/plants14243860

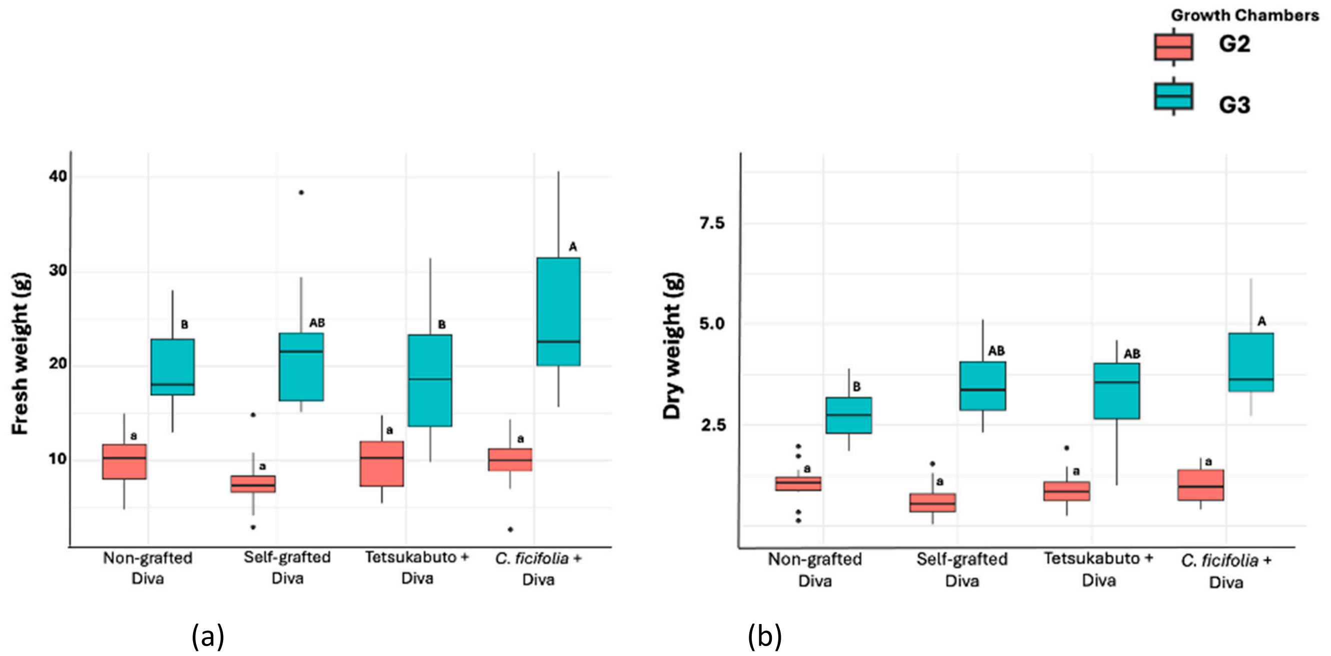

Figure S2: Boxplot with whiskers represent the (a) fresh weight and (b) dry weight of grafted and ungrafted plants at growth chambers G2 (18/12°C), and G3 (24/18°C) after 21 days. Different letters on bars and boxes indicate significant differences between treatments.

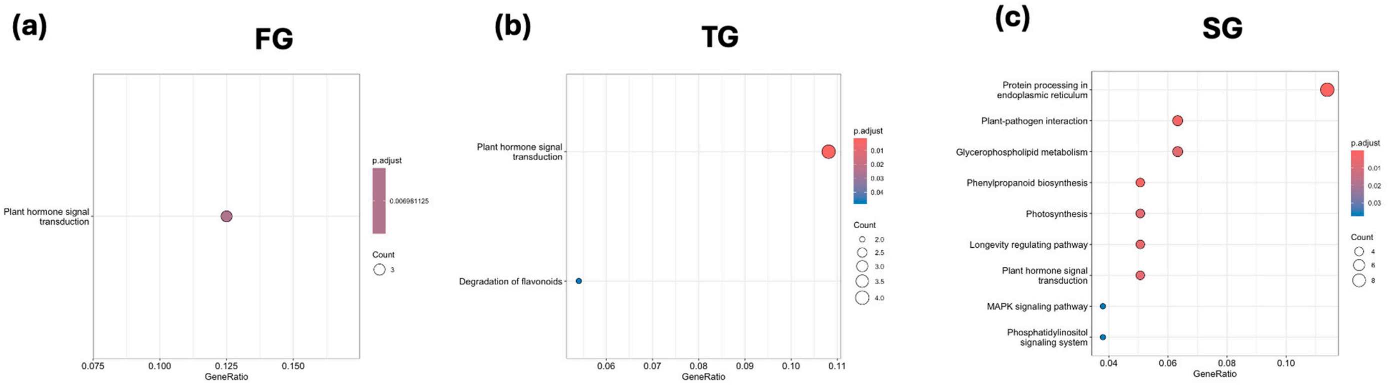

Figure S3: KEGG enrichment analysis of unique DEGs for (a) FG, (b) TG and (c) SG plants at day 0 in growth chamber G2 (18/12°C). FG = cucumber plants grafted onto *C. ficifolia* rootstock, TG = cucumber plants grafted onto Tetsukabuto rootstock and SG = self-grafted cucumber plants.

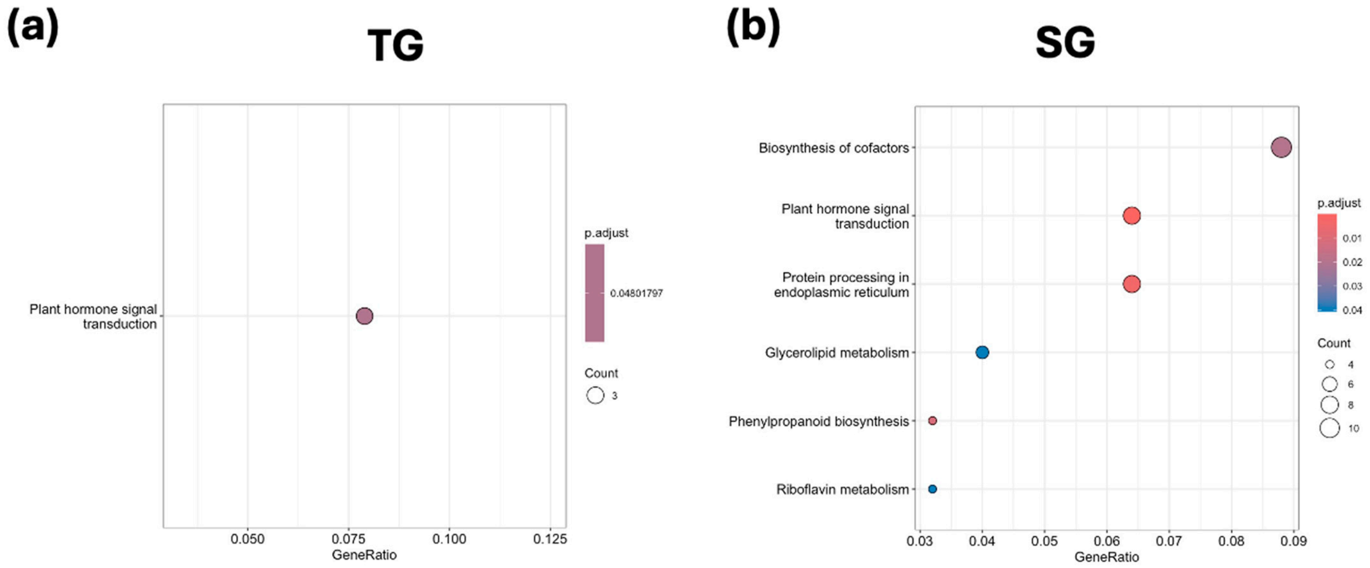

Figure S4: KEGG enrichment analysis of unique DEGs for (a) TG, and (b) SG plants at day 21 in growth chamber G2 (18/12°C). TG = cucumber plants grafted onto Tetsukabuto rootstock and SG = self-grafted cucumber plants.
